# The Influence of AQP5 on the Response to Hydrogen Peroxide in Breast Cancer Cell Lines

**DOI:** 10.3390/ijms26073243

**Published:** 2025-03-31

**Authors:** Ivan Lučić, Monika Mlinarić, Ana Čipak Gašparović, Lidija Milković

**Affiliations:** Laboratory for Membrane Transport and Signaling, Division of Molecular Medicine, Ruđer Bošković Institute, Bijenička Cesta 54, 10000 Zagreb, Croatia; ivan.lucic@irb.hr (I.L.); monika.mlinaric@irb.hr (M.M.)

**Keywords:** AQP5, AQP3, hydrogen peroxide, NRF2 signaling, PI3K/AKT signaling, FOXOs, ROS production, cell viability

## Abstract

Breast cancer is a heterogeneous disease with varying responses to therapies. While targeted treatments have advanced, conventional therapies inducing oxidative stress remain widely used. H_2_O_2_ has emerged as a therapeutic candidate due to its role in signaling and cell-function regulation. Its transport is tightly regulated through peroxiporins such as AQP5, expression of which is linked to poor prognosis and metastatic spread, and its role in therapy resistance remains underexplored. This study examined AQP5’s role in the acute oxidative stress response. We overexpressed AQP5 in breast cancer cell lines with low basal levels—HR+ (MCF7), HER2+ (SkBr-3), and TNBC (SUM 159)—and exposed them to H_2_O_2_ for 24 h. We assessed cell viability, intracellular ROS, changes in AQP3 and AQP5, and key antioxidative and cancer-related pathways (NRF2, PI3K/AKT, FOXOs). AQP5 overexpression elicited a cell-type-specific response. H_2_O_2_ treatment reduced viability in SkBr-3-AQP5 and MCF7-AQP5 cells, increased ROS levels in MCF7-AQP5, and decreased ROS in SUM 159-AQP5. It also increased *AQP3* in MCF7-AQP5 and differentially affected NRF2, FOXOs, and PI3K/AKT signaling, notably activating NRF2/AKR1B10 axis in MCF7-AQP5 and decreasing FOXO1 in SUM 159-AQP5. These findings highlight the need for further research into AQP5’s role in the oxidative stress response in breast cancer cells.

## 1. Introduction

Aquaporins (AQP) are integral cell-membrane proteins that selectively facilitate water transport, with some of them facilitating the transport of glycerol and other small solutes as well [1]. They share a similar structure, a tetrameric assembly of monomers surrounding a central pore. Each monomer acts independently and is composed of six transmembrane α-helices, five connecting loops, and cytoplasmic N- and C-termini, such that the monomers create a pore for transport activity [2]. In humans, there are thirteen known isoforms of AQPs, which are classified based on their primary sequence and permeability into three groups: orthodox aquaporins (AQP0, 1, 2, 4, 5, 6, and 8), aquaglyceroporins (AQP3, 7, 9, and 10), and unorthodox or super aquaporins (AQP11 and 12) [2,3]. Recently, a new subgroup called peroxiporins has emerged. This group is composed of differentially classified AQPs (AQP1, 3, 5, 8, 9, and 11) that facilitate the transport of hydrogen peroxide as well [4].

Hydrogen peroxide (H_2_O_2_) is one of the main reactive oxygen species (ROS) produced during oxidative stress and is known to contribute to cellular signaling pathways [5]. ROS, particularly hydrogen peroxide, affect cell fate in a concentration-dependent manner, with outcomes ranging from proliferation to cell death. As such, they can contribute to both cancer development and resistance to therapy while also playing a crucial role in preventing the growth of transformed cells and in anticancer therapy [6]. The main pathway activated to mitigate oxidative stress is the NRF2 (nuclear factor erythroid 2–related factor 2) pathway. Under basal conditions, NRF2 is repressed by KEAP1 (Kelch-like ECH-associated protein 1) and targeted for ubiquitination. However, during oxidative stress, ROS induce conformational changes in KEAP1, preventing NRF2 degradation. Newly synthesized NRF2 then translocates to the nucleus, where it activates the expression of various antioxidative genes, as well as the expression of genes involved in cellular metabolism [7]. Additionally, Forkhead box class O (FOXO) transcription factors are involved in the antioxidative response to oxidative stress [8]. Both NRF2 and FOXOs exhibit dual roles in cancer, acting as suppressors of malignant transformation while also preventing ROS-driven apoptosis in transformed cells. Additionally, they may contribute to therapy resistance and are linked to the phosphoinositide 3-kinase/protein kinase B (PI3K/AKT) signaling pathway, which is known to be activated in cancer to promote cancer progression [9,10,11,12,13].

Breast cancer is the most common malignancy among women worldwide and constitutes a global health burden [14]. It is a highly heterogeneous disease with diverse classifications. The most common classification is based on its molecular features, specifically the expression of specific receptors—hormone receptors (HR; estrogen (ER) and progesterone receptor (PR)) and human epidermal growth factor receptor 2 (HER2)—and the proliferation marker Ki67, and these features are used to classify breast cancers into luminal A, luminal B, HER2-enriched, and triple-negative (TNBC)/basal-like breast cancer [15]. Each breast cancer subtype responds differently to anticancer therapies, depending on the pathways involved [16] including conventional treatments such as radiotherapy and chemotherapy [17,18]. Regardless of their primary anticancer mechanism, conventional chemo- and radiotherapy elevate intracellular ROS levels, ultimately inducing cancer cell death [19,20].

AQP5 is absent in normal breast tissue but is expressed in breast cancer, where it shows differential expression among subtypes, with higher expression linked to worse prognosis and metastatic spread [21,22]. AQP5 drives breast cancer progression by downregulating the polarity protein Scribble and activating Ras and WNT (Wingless-related integration site)/β-catenin signaling, leading to disrupted cell polarity, enhanced proliferation, epithelial–mesenchymal transition (EMT), and cancer-cell dissemination [23,24]. Additionally, studies on cancer cell lines suggest that AQP5 plays a significant role in the response to chemotherapy and may promote therapy resistance, highlighting AQP5 as a potential therapeutic target [25,26]. In HT-29 colon cancer cells, AQP5 has been shown to contribute to resistance to 5-fluorouracil and cisplatin, as silencing of AQP5 suppressed multidrug-resistance (MDR) genes through inhibition of the p38 mitogen-activated protein kinase (MAPK) pathway [27]. In adriamycin-resistant MCF7 cells, AQP5 silencing enhanced chemosensitivity to doxorubicin [28]. Similarly, overexpression of AQP5 mitigated the reduction in viability of MCF7 spheroids that resulted from treatment with 5-fluorouracil and cisplatin, although these treatments and AQP5 overexpression did not affect the viability of MDA-MB-231 spheroids [29]. Interestingly, AQP5 overexpression increased sensitivity to doxorubicin in both MCF7 and MDA-MB-231 spheroids [29]. While in MCF7 cells, AQP5 acted through the Ras signaling pathway, this was not the case in MDA-MB-231 cells [29]. These findings indicate the need for further research into the role of AQP5 in therapy resistance, emphasizing the importance of considering specific drugs and their effects on different breast cancer subtypes. Additionally, better characterization of the pathways influenced by AQP5 is crucial. As a peroxiporin [30], AQP5 may regulate signaling pathways by modulating H_2_O_2_ levels. Consistent with this hypothesis, our findings demonstrate that oxidative stress differentially modulates AQP5 and NRF2 expression in breast cancer cell lines with varying degrees of malignancy [31].

In this study, we aimed to further investigate the role of AQP5 in the acute response of breast cancer cell lines to oxidative stress. We utilized breast cancer cell lines representing different subtypes and degrees of malignancy—HR+ (MCF7), HER2+ (SkBr-3), and TNBC (SUM 159)—all of which exhibit low basal levels of AQP5 mRNA [32]. To assess the effects of AQP5, we transfected these cells with a plasmid encoding human *AQP5* and exposed them to hydrogen peroxide for 24 h. We evaluated cell viability, intracellular ROS production, and the involvement of major signaling pathways implicated in the antioxidative response and cancer progression (the NRF2, PI3K/AKT, and FOXO pathways). Additionally, we assessed whether the expression of another peroxiporin, AQP3, is altered in response to AQP5 overexpression.

## 2. Results

### 2.1. Overexpression of AQP5 Decreases Cell Viability in MCF7 and SkBr-3 Cells Following Treatment with 40 µM Hydrogen Peroxide

**Figure 1:**
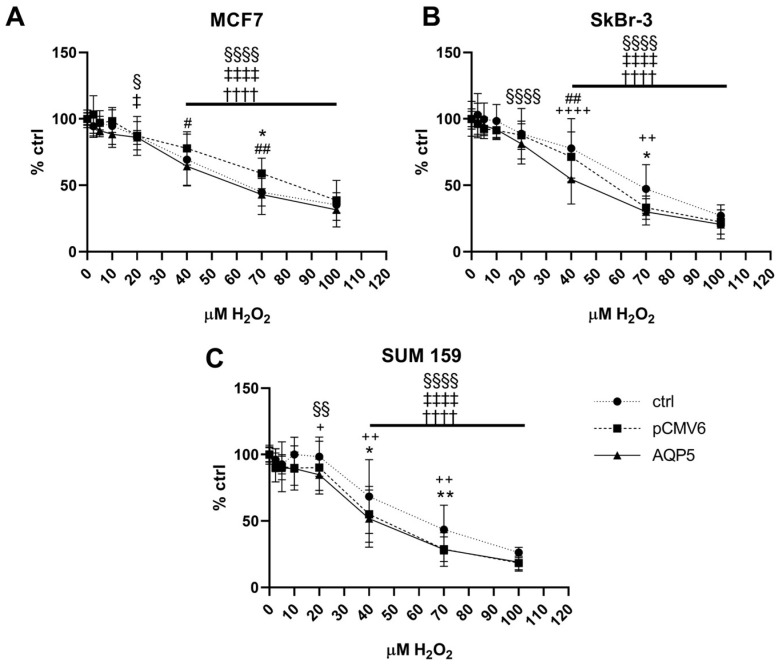
Effect of AQP5 overexpression and 24-h treatment with hydrogen peroxide on the viability of breast cancer cell lines: (**A**) MCF7 cell line; (**B**) SkBr-3 cell line; (**C**) SUM 159 cell line. Cell lines were either non-transfected (control denoted as ctrl) or transfected with plasmid encoding human *AQP5* (denoted as AQP5) or empty plasmid (denoted as pCMV6). The results are expressed as means ± SDs, *n* = 5. Significance markers (†, ‡, §) represent differences between H_2_O_2_-treated cells and their respective controls (untreated cells) for: †—control (non-transfected) cells, ‡—pCMV6 cells, §—AQP5-overexpressing cells. (*) indicates differences between control (non-transfected) vs. pCMV6 cells for the same treatment. (#) indicates differences between pCMV6 vs. AQP5-overexpressing cells for the same treatment. (+) indicates differences between control (non-transfected) vs. AQP5-overexpressing cells for the same treatment. Significance levels: ‡, §, *, +, # for *p* < 0.05; §§, **, ++, ## for *p* < 0.01; ††††, ‡‡‡‡, §§§§, ++++ for *p* < 0.0001. Abbreviations: H_2_O_2_, hydrogen peroxide.

To evaluate the effects of hydrogen peroxide on cell viability, we exposed control cells and stably transfected breast cancer cell lines (MCF7, SkBr-3, and SUM 159) to increasing concentrations of hydrogen peroxide for 24 h. Cells were transfected with either a plasmid encoding human *AQP5* or an empty plasmid (pCMV6). Cell viability significantly decreased at hydrogen peroxide concentrations of 40 µM and higher (*p* < 0.0001; Figure 1A–C). Breast cancer cell lines overexpressing AQP5 (AQP5 cells) showed greater sensitivity to hydrogen peroxide, with a significant decrease in viability observed at 20 µM (MCF7-AQP5: *p* = 0.0205; SkBr-3-AQP5: *p* < 0.0001; SUM 159-AQP5: *p* = 0.0022). Interestingly, a similar effect was observed only in MCF7-pCMV6 cells (*p* = 0.0353).

Further, we were interested in how AQP5 affects cell viability across different breast cancer cell lines. High AQP5 expression significantly reduced the viability of MCF7 cells compared to pCMV6 cells following exposure to 40 µM and 70 µM hydrogen peroxide (*p* = 0.0242 and *p* = 0.0049, respectively; Figure 1A). Similarly, SkBr-3-AQP5 cells showed decreased viability after treatment with 40 µM hydrogen peroxide compared to pCMV6 cells and control cells (*p* = 0.0023 and *p* < 0.0001, respectively; Figure 1B). Both SkBr-3-pCMV6 and Skbr-3-AQP5 were more sensitive to 70 µM hydrogen peroxide than were control cells (*p* = 0.0449 and *p* = 0.0011). On the other hand, there was no difference in viability between SUM 159-AQP5 and SUM 159-pCMV6 cells (Figure 1C). However, they were both more sensitive to treatment with 40 µM and 70 µM hydrogen peroxide compared to control cells (*p* = 0.0019, *p* = 0.0135, *p* = 0.0052, and *p* = 0.0044, respectively).

### 2.2. Overexprexpression of AQP5 Increases Intracellular Levels of ROS in MCF7 Cells While Decreasing ROS Levels in SUM 159 Cells Following Hydrogen Peroxide Treatment

**Figure 2:**
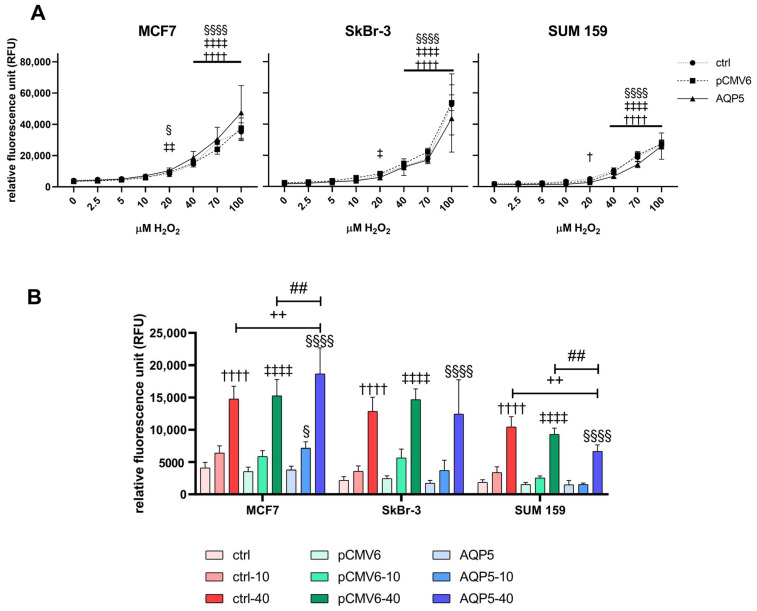
Effect of the AQP5 overexpression on the intracellular ROS generation in the breast cancer cell lines. (**A**) Intracellular ROS levels upon a range of H_2_O_2_ concentrations in MCF7, SkBr-3, and SUM 159 cells. (**B**) Intracellular ROS levels upon the addition of selected H_2_O_2_ concentrations (10 and 40 µM). Cell lines were either non-transfected (control denoted as ctrl) or transfected with plasmid encoding human *AQP5* (denoted as AQP5) or empty plasmid (denoted as pCMV6). The results are expressed as means ± SDs, *n* = 3. Significance markers (†, ‡, §) represent differences between H_2_O_2_-treated cells and their respective controls (untreated cells) for: †—control (non-transfected) cells, ‡—pCMV6 cells, §—AQP5-overexpressing cells. (#) indicates differences between pCMV6 vs. AQP5-overexpressing cells for the same treatment. (+) indicates differences between control (non-transfected) vs. AQP5-overexpressing cells for the same treatment. Significance levels: †, ‡, § for *p* < 0.05; ‡‡, ++, ## for *p* < 0.01; ††††, ‡‡‡‡, §§§§ for *p* < 0.0001. Abbreviations: ROS, Reactive oxygen species; H_2_O_2_, hydrogen peroxide.

Since AQP5 is a peroxiporin, we were interested in whether overexpression of AQP5 affects the intracellular generation of ROS upon the exogenous addition of a range of H_2_O_2_ concentrations (0, 2.5, 5, 10, 20, 40, 70, and 100 µM) in the tested breast cancer cell lines. A significant increase in ROS production started in all cell lines upon the addition of 40 µM H_2_O_2_ (Figure 2A). However, the addition of 20 µM H_2_O_2_ partially affected ROS generation, resulting in an increase of ROS in MCF7-pCMV6 (*p* = 0.0065), MCF7-AQP5 (*p* = 0.0174), SkBr-3-pCMV6 (*p* = 0.0379), and SUM 159-ctrl cells (*p* = 0.0157). SUM 159 cells showed the lowest levels of ROS upon H_2_O_2_ challenge (Figure 2A).

We further selected two concentrations of H_2_O_2_: 10 µM, which did not affect cell viability, and 40 µM, which was the lowest concentration affecting cell viability and intracellular ROS production, and evaluated the differences between the cell lines tested. We observed that 40 µM H_2_O_2_ significantly increases intracellular ROS levels within 15 min of its addition (Figure 2B and Figure S2) and that this effect continues over time in all cells.

Interestingly, AQP5 differentially impacted ROS levels in breast cancer cell lines. In MCF7 cells, upon challenge with 40 µM H_2_O_2_, MCF7-AQP5 cells showed significantly increased intracellular ROS (*p* < 0.01) compared to both control and pCMV6 cells. Conversely, SUM 159-AQP5 cells showed decreased ROS levels (*p* < 0.01; Figure 2B).

### 2.3. Overexpression of AQP5 Increases the RNA Expression of AQP3 in MCF7 Cells in Response to Hydrogen Peroxide

**Figure 3:**
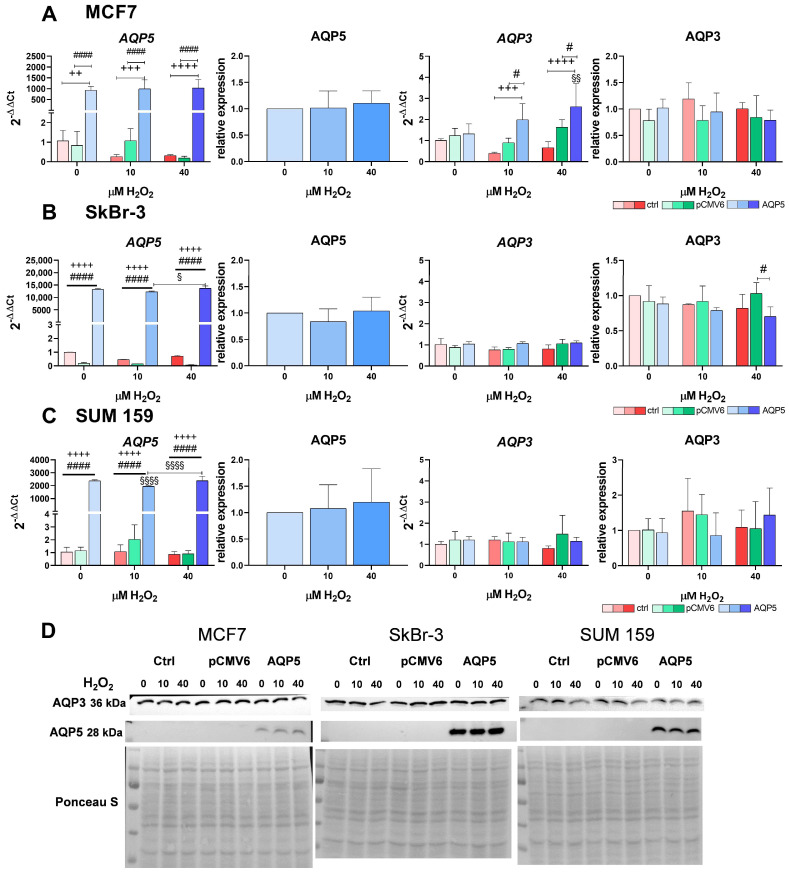
AQP5 and AQP3 mRNA and protein expression in breast cancer cell lines: (**A**) MCF7, (**B**) SkBr-3, (**C**) SUM 159, and (**D**) representative western blots. Cell lines were either non-transfected (control denoted as ctrl, shown in shades of red with increasing intensity as H_2_O_2_ concentration rises) or transfected with plasmid encoding human *AQP5* (denoted as AQP5, shown in shades of blue with increasing intensity as H_2_O_2_ concentration rises) or empty plasmid (denoted as pCMV6, shown in shades of green with increasing intensity as H_2_O_2_ concentration rises). The results are expressed as means ± SDs, *n* = 3. Significance markers (§) represent differences between H_2_O_2_-treated and untreated (control) AQP5-overexpressing cells. (#) indicates differences between pCMV6 vs. AQP5-overexpressing cells for the same treatment. (+) indicates differences between control (non-transfected) vs. AQP5-overexpressing cells for the same treatment. Significance levels: §, # for *p* < 0.05; §§, ++ for *p* < 0.01; +++ for *p* < 0.001; §§§§, ++++, #### for *p* < 0.0001. Abbreviations: AQP3, aquaporin 3; AQP5, aquaporin 5; H_2_O_2,_ hydrogen peroxide.

The overexpression of AQP5 was confirmed in all breast cancer cell lines at both the RNA and protein levels (Figure 3A–D). Among them, SkBr-3-AQP5 cells showed the highest levels of AQP5 expression (Figure 3B,D and Figure S3). Treatment with H_2_O_2_ influenced the mRNA expression of *AQP5* in SkBr-3-AQP5 and SUM 159-AQP5 cells (Figure 3B,C). Specifically, 10 µM H_2_O_2_ significantly decreased *AQP5* expression in SUM 159-AQP5 cells compared to the untreated control (*p* < 0.0001). However, increasing the concentration to 40 µM H_2_O_2_ restored *AQP5* expression in SUM 159-AQP5 (10 vs. 40 µM H_2_O_2_ *p* < 0.0001). A similar trend was observed in SkBr-3-AQP5 cells (10 vs. 40 µM H_2_O_2_ *p* = 0.0139).

Furthermore, the overexpression of AQP5 and H_2_O_2_ treatment influenced *AQP3* expression exclusively in MCF7-AQP5 cells (Figure 3A,D). Exposure to both 10 and 40 µM H_2_O_2_ resulted in significantly greater *AQP3* expression in MCF7-AQP5 cells compared to that in MCF7 and MCF7-pCMV6 cells (*p* < 0.001 and *p* < 0.05, respectively). Additionally, *AQP3* expression was significantly higher in MCF7-AQP5 cells treated with 40 µM H_2_O_2_ than in untreated MCF7-AQP5 cells (*p* = 0.0027). However, this increase was not observed at the protein level. A link between AQP5 and AQP3 in SkBr-3 cells was observed only with treatment with 40 µM H_2_O_2_, which resulted in decreased AQP3 protein levels in SkBr-3-AQP5 cells compared to SkBr-3-pCMV6 cells (*p* = 0.0145).

### 2.4. Overexpression of AQP5 Slightly Affects the NRF2 Signaling Pathway—Mainly the NRF2/AKR1B10 Axis in MCF7 Cells upon H_2_O_2_ Treatment

**Figure 4:**
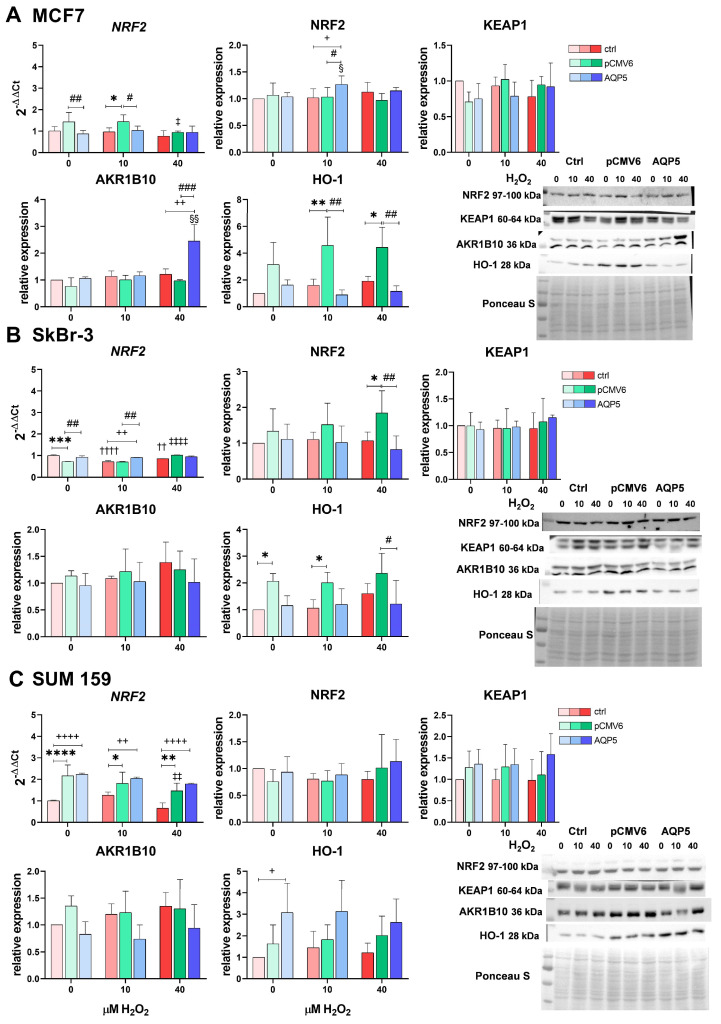
Effects of the AQP5 and hydrogen peroxide treatment on the NRF2 signaling pathway: mRNA expression of NRF2 and protein expression of NRF2, KEAP1, AKR1B10, and HO-1 in breast cancer cell lines: (**A**) MCF7, (**B**) SkBr-3, and (**C**) SUM 159. Cell lines were either non-transfected (control denoted as ctrl, shown in shades of red with increasing intensity as H_2_O_2_ concentration rises) or transfected with plasmid encoding human *AQP5* (denoted as AQP5, shown in shades of blue with increasing intensity as H_2_O_2_ concentration rises) or empty plasmid (denoted as pCMV6, shown in shades of green with increasing intensity as H_2_O_2_ concentration rises). The results are expressed as means ± SDs, *n* = 3. Significance markers (†, ‡, §) represent differences between H_2_O_2_-treated cells and their respective controls (untreated cells) for: †—control (non-transfected) cells, ‡—pCMV6 cells, §—AQP5-overexpressing cells. (*) indicates differences between control (non-transfected) vs. pCMV6 cells for the same treatment. (#) indicates differences between pCMV6 vs. AQP5-overexpressing cells for the same treatment. (+) indicates differences between control (non-transfected) vs. AQP5-overexpressing cells for the same treatment. Significance levels: ‡, §, *, +, # for *p* < 0.05; ††, ‡‡, §§, **, ++, ## for *p* < 0.01; ***, ### for *p* < 0.001; ††††, ‡‡‡‡, ****, ++++ for *p* < 0.0001. Abbreviations: NRF2, nuclear factor erythroid 2–related factor 2; KEAP1, Kelch-like ECH-associated protein 1; HO-1, Heme oxygenase 1; AKR1B10, Aldo-keto reductase family 1 member B10.

The NRF2 signaling pathway is the main pathway activated as a response to oxidative stress. Consequently, we investigated whether overexpression of AQP5 influences the NRF2 signaling pathway upon H_2_O_2_ treatment. We analyzed the RNA expression of *NFE2L2 (NRF2)* by qPCR and its protein levels by western blot, which was also used to quantify protein levels of the NRF2 repressor KEAP1 and the downstream targets aldo-keto reductase family 1 member B10 (AKR1B10) and heme oxygenase 1 (HO-1). We observed a cell-line-specific response (Figure 4A–C and S3).

In the MCF7 cell line (Figure 4A), AQP5 increased protein levels of NRF2 following treatment with 10 µM H_2_O_2_ (*p* = 0.0255 vs. its control; *p* = 0.0112 vs. MCF7; and *p* = 0.0195 vs. MCF7-pCMV6) and increased levels of its downstream target AKR1B10 following treatment with 40 µM H_2_O_2_ (*p* = 0.0012 vs. its control; *p* = 0.0028 vs. MCF7; and *p* = 0.0008 vs. MCF7-pCMV6). However, transfection with the pCMV6 vector elevated *NRF2* expression compared to both control and MCF7-AQP5 cells (*p* < 0.05). This increase was reduced upon treatment with 40 µM H_2_O_2_ (*p* = 0.0275). A similar trend was observed for HO-1 protein levels, with MCF7-pCMV6 cells showing higher levels than the control and MCF7-AQP5 cells following treatments with 10 and 40 µM H_2_O_2_ (*p* < 0.05).

In SkBr-3 cells (Figure 4B), AQP5 overexpression had a modest effect on NRF2 signaling. Specifically, AQP5 increased *NRF2* expression following treatment with 10 µM H_2_O_2_ (*p* = 0.003 vs. MCF7 and *p* = 0.0023 vs. MCF7-pCMV6) but did not affect NRF2 protein levels, levels of the downstream targets, or the levels of its repressor KEAP1. Additionally, the pCMV6 vector decreased basal *NRF2* expression compared to control, MCF7 (*p* = 0.0002), and MCF7-AQP5 cells (*p* = 0.0019), which increased following 40 µM H_2_O_2_ (*p* < 0.0001 vs. untreated MCF7-pCMV6). Correspondingly, NRF2 protein levels also increase following 40 µM H_2_O_2_ (*p* = 0.0223 vs. MCF7 and *p* = 0.0013 vs. MCF7-AQP5). Similar to MCF7 cells, HO-1 protein levels in SkBr-3 cells were influenced by pCMV6 transfection, showing higher levels compared to MCF7 and MCF7-AQP5 cells (*p* < 0.05). Additionally, H_2_O_2_ treatments decreased *NRF2* expression in MCF7 cells (vs. untreated *p* < 0.001).

In SUM 159 cells, transfection generally led to increased *NRF2* mRNA expression in both SUM 159-pCMV6 cells and SUM 159-AQP5 cells compared to control SUM 159 cells regardless of the H_2_O_2_ treatment (*p* < 0.05; Figure 3C). However, we did observe higher expression of its downstream target HO-1 in SUM 159-AQP5, with a significant increase compared to control SUM 159 in the untreated condition (*p* = 0.0162). Additionally, treatment with 40 µM H_2_O_2_ reduced *NRF2* mRNA expression in SUM 159-pCMV6 cells (*p* = 0.0053). Although similar trends were observed in control and AQP5-overexpressing SUM 159 cells, these changes were not statistically significant.

### 2.5. AQP5 Overexpression Decreases Total AKT Levels in MCF7-AQP5 and SkBr-3-AQP5 Cells and Increases PTEN Levels in SUM 159-AQP5 Cells Following Treatment with 40 µM H_2_O_2_

The PI3K/AKT signaling pathway promotes tumor-cell proliferation and survival and cancer progression. Since AQP5 has been suggested to play a role in breast cancer cell proliferation, we investigated whether AQP5 overexpression affects PI3K/AKT signaling.

**Figure 5:**
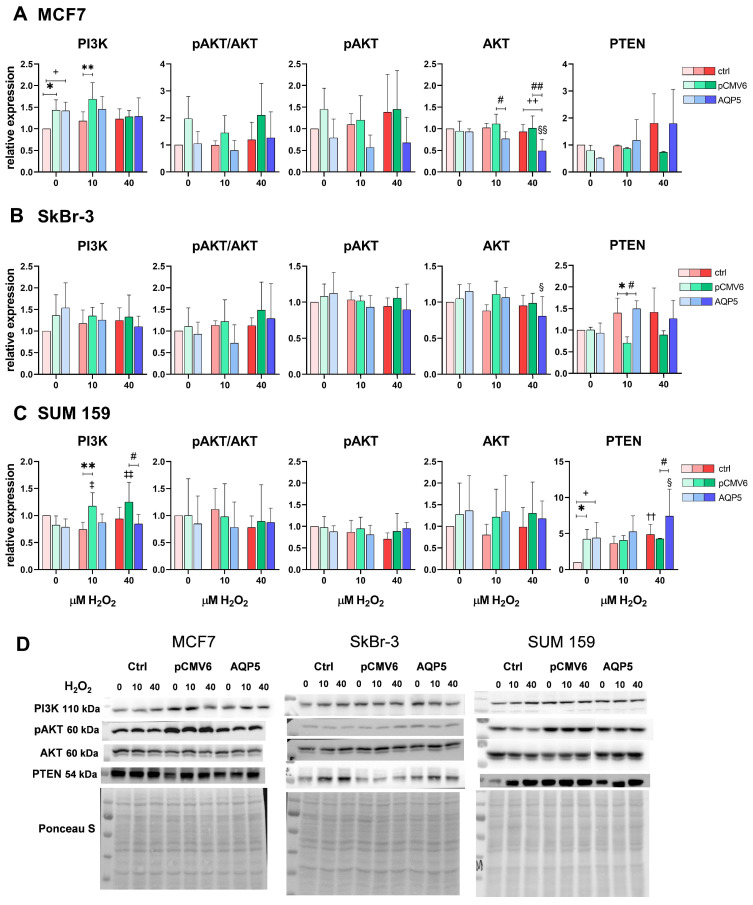
Effects of the AQP5 and hydrogen peroxide treatment on the PI3K/AKT signaling pathway in breast cancer cell lines: (**A**) MCF7, (**B**) SkBr-3, (**C**) SUM 159, and (**D**) representative western blots. Cell lines were either non-transfected (control denoted as ctrl, shown in shades of red with increasing intensity as H_2_O_2_ concentration rises) or transfected with plasmid encoding human *AQP5* (denoted as AQP5, shown in shades of blue with increasing intensity as H_2_O_2_ concentration rises) or empty plasmid (denoted as pCMV6, shown in shades of green with increasing intensity as H_2_O_2_ concentration rises). The results are expressed as means ± SDs, *n* = 3. Significance markers (†, ‡, §) represent differences between H_2_O_2_-treated cells and their respective controls (untreated cells) for: †—control cells (non-transfected), ‡—pCMV6 cells, §—AQP5-overexpressing cells. (*) indicates differences between control (non-transfected) vs. pCMV6 cells for the same treatment. (#) indicates differences between pCMV6 vs. AQP5-overexpressing cells for the same treatment. (+) indicates differences between control (non-transfected) vs. AQP5-overexpressing cells for the same treatment. Significance levels: ‡, §, *, +, # for *p* < 0.05; ††, ‡‡, §§, **, ++, ## for *p* < 0.01. Abbreviations: PI3K, Phosphoinositide 3-kinases; pAKT, phosphorylated protein kinase B; AKT, protein kinase B; PTEN, Phosphatase and tensin homolog.

In all three tested breast cancer cell lines, AQP5 overexpression did not activate the PI3K/AKT signaling pathway, as evidenced by the lack of an increase in phosphorylated AKT or the pAKT/AKT ratio (Figure 5A–C). Interestingly, AQP5 reduced total AKT expression in SkBr-3-AQP5 cells following treatment with 40 µM H_2_O_2_ (*p* = 0.0127) and particularly in MCF7-AQP5 cells (*p* = 0.0078) compared to control MCF7 and MCF7-pCMV6 cells (*p* = 0.0079 and *p* = 0.0015, respectively). In MCF7 cells, AQP5-mediated decreases in total AKT levels compared to those in MCF7-pCMV6 cells (*p* = 0.0398) were also observed following treatment with 10 µM H_2_O_2_.

Additionally, transfected but untreated MCF7-pCMV6 cells and MCF7-AQP5 cells exhibited higher PI3K expression than did control MCF7 cells (*p* = 0.0113 and *p* = 0.0146, respectively). Higher PI3K expression persisted in MCF7-pCMV6 cells following treatment with 10 µM H_2_O_2_ than in control MCF7 cells (*p* = 0.0025). In SUM 159 cells, transfection and hydrogen peroxide treatments increased PI3K levels, but only in SUM 159-pCMV6 cells (vs. untreated SUM 159-pCMV6 *p* = 0.0285 and *p* = 0.007; vs. SUM 159 *p* = 0.0058). In these cells, PI3K levels increased upon transfection with the pCMV6 vector and were decreased by AQP5 following treatment with 40 µM H_2_O_2_ (*p* = 0.0161).

Moreover, upon treatment with 40 µM H_2_O_2_, AQP5 increased the levels of phosphatase and tensin homolog (PTEN), an inhibitor of the PI3K/AKT signaling pathway in SUM 159-AQP5 cells (vs. its untreated control, *p* = 0.0348 and vs. SUM 159-pCMV6 cells, *p* = 0.0395). Similarly, 40 µM H_2_O_2_ increased PTEN levels in control SUM 159 cells, but this effect was not observed in SUM 159-pCMV6 cells. However, transfection generally resulted in elevated PTEN levels compared to those observed in control SUM 159 cells (vs. SUM 159-pCMV6, *p* = 0.0241 and vs. SUM 159-AQP5, *p* = 0.0168).

Conversely, in SkBr-3 cells treated with 10 µM H_2_O_2_, PTEN levels decreased in SkBr-3-pCMV6 cells (mock control) but were restored by AQP5 to levels comparable to those observed in control SkBr-3 cells, which also exhibited higher PTEN expression than SkBr-3-pCMV6 cells (*p* = 0.0218 and *p* = 0.0245, respectively).

### 2.6. Overexpression of AQP5 and Hydrogen Peroxide Treatment Slightly Affect FOXO1 and FOXO3a, Primarily Decreasing FOXO1 Protein Levels in SUM 159 Cells and Increasing FOXO3a mRNA Expression in SkBr-3 Cells

FOXO family transcription factors are important in the oxidative stress response, as they induce the expression of antioxidant enzymes, including superoxide dismutase, catalase, and peroxiredoxins, which eliminate ROS. In addition, they regulate processes such as the cell cycle, apoptosis, and metabolism. Although FOXOs are predominantly recognized as tumor suppressors, evidence suggests they may contribute to cancer growth under certain conditions. Their role in breast cancer is closely linked to PI3K/AKT signaling. FOXO1 and FOXO3a are among the most studied FOXO transcription factors and have been shown to have significant roles in breast cancer progression and regulation. Therefore, we investigated whether AQP5 overexpression and hydrogen peroxide treatment influence their mRNA and protein expression.

**Figure 6:**
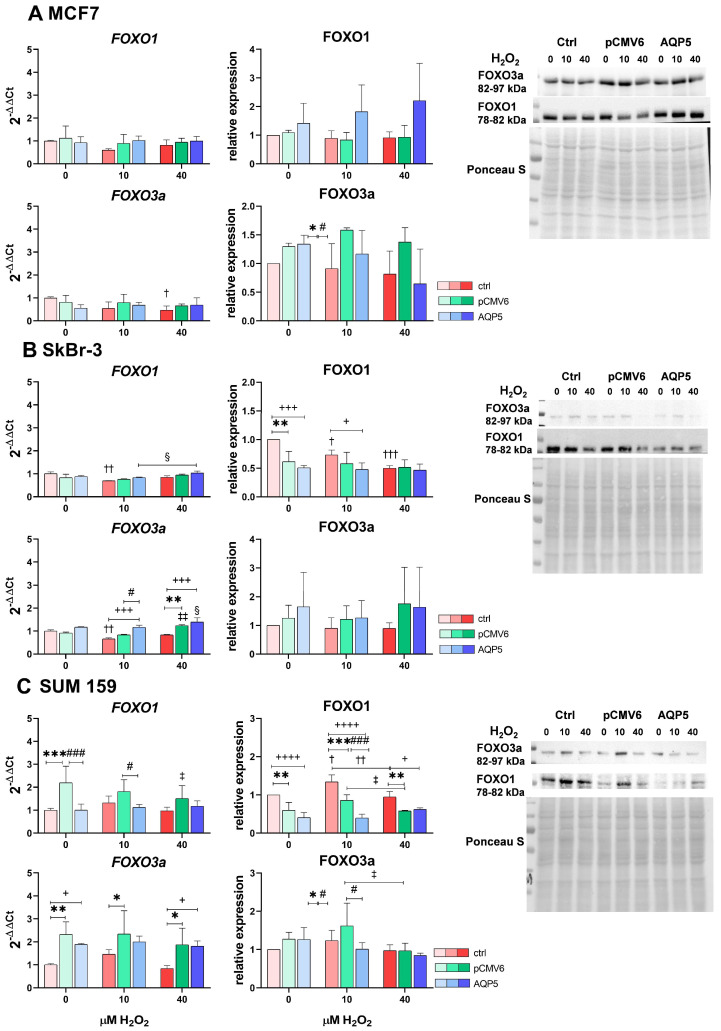
Effects of the AQP5 and hydrogen peroxide treatment on the FOXO1 and FOXO3a mRNA and protein expression in breast cancer cell lines: (**A**) MCF7, (**B**) SkBr-3, (**C**) SUM 159. Cell lines were either non-transfected (control denoted as ctrl, shown in shades of red with increasing intensity as H_2_O_2_ concentration rises) or transfected with plasmid encoding human *AQP5* (denoted as AQP5, shown in shades of blue with increasing intensity as H_2_O_2_ concentration rises) or empty plasmid (denoted as pCMV6, shown in shades of green with increasing intensity as H_2_O_2_ concentration rises). The results are expressed as means ± SDs, *n* = 3. Significance markers (†, ‡, §) represent differences between H_2_O_2_-treated cells and their respective controls (untreated cells) for: †—control (non-transfected) cells, ‡—pCMV6 cells, §—AQP5-overexpressing cells. (*) indicates differences between control (non-transfected) vs. pCMV6 cells for the same treatment. (#) indicates differences between pCMV6 vs. AQP5-overexpressing cells for the same treatment. (+) indicates differences between control (non-transfected) vs. AQP5-overexpressing cells for the same treatment. Significance levels: †, ‡, §, *, +, # for *p* < 0.05; ††, ‡‡, ** for *p* < 0.01; †††, ***, +++, ### for *p* < 0.001; ++++ for *p* < 0.0001. Abbreviations: FOXO1, forkhead box class O 1; FOXO3a, forkhead box class O 3a.

We observed a breast cancer cell-line-specific response. In MCF7 cells (Figure 6A), AQP5 did not affect the protein and mRNA expression of FOXO1 and FOXO3a, although a slight, non-statistically significant increase in FOXO1 levels was observed. Additionally, in control MCF7 cells, 40 µM H_2_O_2_ significantly decreased levels of *FOXO3a* (*p* = 0.0320).

Among SkBr-3 cells (Figure 6B), control SkBr-3 cells had higher basal protein levels of FOXO1 than did SkBr-3-pCMV6 and SkBr-3-AQP5 cells (*p* = 0.0021 and *p* = 0.0002). Treatment with 10 µM H_2_O_2_ decreased mRNA and protein levels of FOXO1 in control SkBr-3 cells (*p* = 0.0059 and *p* = 0.03), while 40 µM H_2_O_2_ further reduced protein levels of FOXO1 (*p* = 0.0002) to the levels observed in SkBr-3-pCMV6 and SkBr-3-AQP5 cells. While we did not observe such an effect on protein level, we did observe an increase in levels of *FOXO1* with increasing concentrations of hydrogen peroxide in SkBr-3-AQP5 cells (*p* = 0.0476). Similarly, 10 µM H_2_O_2_ decreased levels of *FOXO3a* mRNA in control SkBr-3 cells, while AQP5 increased *FOXO3a* levels compared to those observed in both controls (*p* = 0.0006 and *p* = 0.0140). Treatment with 40 µM H_2_O_2_ further increased *FOXO3a* levels in SkBr-3-AQP5 cells (vs. untreated SkBr-3-AQP5 *p* = 0.0342 and vs. SkBr-3 *p* = 0.0003) while also increasing it in SkBr-3-pCMV6 cells (vs. untreated SkBr-3-pCMV6 *p* = 0.0049 and vs. SkBr-3 *p* = 0.0036).

In SUM 159 cells (Figure 6C), transfection affected the mRNA levels of *FOXO1* and *FOXO3a*, but these changes did not correlate with their protein levels. Specifically, basal *FOXO1* mRNA levels were higher in SUM 159-pCMV6 cells compared to control SUM 159 and SUM 159-AQP5 cells (*p* = 0.0005 for both). This increase persisted upon treatment with 10 µM H_2_O_2_, as seen in the comparison to SUM 159-AQP5 cells (*p* = 0.0473), suggesting that AQP5 reduces *FOXO1* levels to those of control SUM 159 cells. However, 40 µM H_2_O_2_ reduced *FOXO1* mRNA levels in SUM 159-pCMV6 cells (*p* = 0.0489). At the protein level, FOXO1 levels were highest in control SUM 159 cells. Under basal conditions, SUM 159-pCMV6 and SUM 159-AQP5 cells exhibited lower FOXO1 protein levels than did control SUM 159 cells (*p* = 0.0031 and *p* < 0.0001, respectively). In control cells, treatment with 10 µM H_2_O_2_ significantly increased FOXO1 protein levels (*p* = 0.0112), while 40 µM H_2_O_2_ reduced them to basal levels (*p* = 0.0039). A similar trend was observed in SUM 159-pCMV6 cells, where 10 µM H_2_O_2_ slightly increased FOXO1 protein levels; this increase was followed by a decrease on treatment with 40 µM H_2_O_2_ (*p* = 0.0363). In SUM 159-AQP5 cells, treatment with 10 µM H_2_O_2_ resulted in decreased FOXO1 protein levels compared to those observed in control SUM 159 and SUM 159-pCMV6 cells (*p* < 0.0001 and *p* = 0.0008, respectively), while treatment with 40 µM H_2_O_2_ increased them to mock-control levels, which remained lower than those in control SUM 159 cells (vs. SUM 159-pCMV6, *p* = 0.0058; vs. SUM 159-AQP5, *p* = 0.0134).

Both SUM 159-pCMV6 and SUM 159-AQP5 cells showed higher basal mRNA levels of *FOXO3a*, and these levels were largely unaffected by hydrogen peroxide treatment (*p* < 0.05). Protein levels of FOXO3a in SUM 159-pCMV6 cells were influenced by hydrogen peroxide, with a slight increase after treatment with 10 µM H_2_O_2_ and a decrease following treatment with 40 µM H_2_O_2_ (*p* = 0.0212). As observed for FOXO1, AQP5 decreased FOXO3a protein levels following treatment with 10 µM H_2_O_2_ (*p* = 0.0322).

## 3. Discussion

Despite advances in therapeutic approaches that have improved breast cancer management, it remains one of the most prevalent malignancies among women worldwide. The challenges associated with treatment have prompted us to seek potential prognostic and predictive biomarkers, as well as a deeper understanding of the mechanisms underlying treatment responses. Research has shown that each breast cancer subtype responds differently to chemotherapy and radiotherapy and that there are differences within subtypes [18,33].

The high metabolic demands of cancer cells result in elevated levels of ROS, a vulnerability that many conventional treatments, such as radiotherapy and numerous chemotherapeutic agents, exploit. ROS generation serves as a key mechanism of action in radiation therapy and plays a significant role in the efficacy of various chemotherapeutic drugs. As a result, ROS, particularly H_2_O_2_, have emerged as promising candidates for novel therapeutic strategies aimed at selectively enhancing their levels, thereby improving treatment efficacy while minimizing side effects [34]. Because the effects of H_2_O_2_ are highly concentration-dependent, its levels must be tightly regulated within the cell. Endogenous antioxidants are essential for maintaining this balance, with some showing dichotomous effects, while the overexpression of others may contribute to therapy resistance [35,36].

Certain aquaporins, such as AQP5, play a key role in the precise regulation of H_2_O_2_ transport. However, their function extends beyond transporting H_2_O_2_, water, and other small molecules. For instance, AQP5 can interact with the junction proteins, thus affecting cell−cell adhesion [37], and bind to the SH3-domain of members of the Src kinase family (c-Src, Lyn) and adaptor protein Grap2, hence promoting invasion [38]. AQP5 has been implicated in the carcinogenesis of various cancers, including breast cancer, where it contributes to proliferation, migration, and metastasis. In breast cancer, AQP5 downregulates the polarity protein Scribble and activates Ras and WNT/β-catenin signaling, contributing to EMT and cancer-cell dissemination [23,24]. Its expression varies among breast cancer subtypes, with high levels associated with poorer prognosis and increased metastatic potential [21]. Additionally, AQP5 has been implicated in chemotherapy resistance. It has been shown that AQP5 silencing increases sensitivity to cisplatin and 5-FU in HT-29 colon cancer cells by inhibiting the p38 MAPK pathway and suppressing MDR genes [27]. It has also been associated with chemoresistance in MCF7 breast cancer cells [29]. Notably, AQP5 silencing enhances sensitivity to doxorubicin in adriamycin-resistant MCF7 cells [28]. Consequently, AQP5 has been suggested as a potential diagnostic and prognostic biomarker and as a therapeutic target for inhibiting breast cancer progression [39]. However, contrary to expectations, AQP5 overexpression has been linked to increased sensitivity to doxorubicin in MCF7 and MDA-MB-231 spheroids, with that sensitivity arising through diverse mechanisms [29]. Therefore, a deeper understanding of AQP5’s role in breast cancer carcinogenesis and of the underlying mechanisms and conditions influencing its effects is essential.

Here, we aimed to investigate the role of AQP5 in the acute response of breast cancer cell lines to oxidative stress. To achieve this, three breast cancer cell lines, each with low basal levels of AQP5 [32], were stably transfected with either a plasmid encoding human *AQP5* or a mock control (pCMV6 vector). These cell lines represent distinct molecular subtypes of breast cancer: HR+ (MCF7), HER2+ (SkBr-3), and TNBC (SUM 159). Overexpression of AQP5 was successful, and levels were extremely high, as confirmed by qPCR and western blot in all breast cancer cell lines. Among them, SkBr-3-AQP5 cells showed the highest levels of AQP5 expression. Further, we exposed AQP5-overexpressing cells, cells transfected with the empty vector, and control cells from each breast cancer cell line to H_2_O_2_ for 24 h and evaluated their viability, intracellular ROS levels, and the involvement of major signaling pathways related to the antioxidative response and cancer progression (NRF2, PI3K/AKT, and FOXO pathways).

Overexpression of AQP5 increased sensitivity to H_2_O_2_, with significant reductions in cell viability observed in MCF7-AQP5, SkBr-3-AQP5, and SUM 159-AQP5 cells at lower concentrations of H_2_O_2_ (20 µM) compared to their respective controls. Further, starting from 40 µM H_2_O_2_, AQP5 overexpression significantly decreased the viability of SkBr-3 in comparison to both controls and that of MCF7 in comparison to the mock control, which showed greater viability than control cells. In contrast, both SUM 159-AQP5 and SUM 159-pCMV6 exhibited reduced viability compared to the control cells, suggesting that the negative effect is due to transfection and not AQP5. Evaluation of intracellular ROS levels revealed a significant increase upon exposure to 40 µM H_2_O_2_, with differences observed between the cell lines. Given that regulation of cell fate depends on the signaling properties of H_2_O_2_, which are concentration-dependent [6], we selected 10 µM H_2_O_2_, which did not affect cell viability, and 40 µM H_2_O_2_, which reduced viability to varying degrees across all tested cell lines (ranging from IC25–IC50) without causing complete cell death, while also increasing intracellular ROS levels. These concentrations ensured exposure to mild to moderate oxidative stress without excessive toxicity, allowing us to assess differences between the cell lines and investigate whether and how AQP5 influences pathways involved in protection against and adaptation to oxidative stress, as well as cancer progression, all of which are known to be affected by H_2_O_2_ [40].

In all cells, 40 µM H_2_O_2_ significantly increased intracellular ROS levels within 15 min of its addition, and this increase continued over time. Interestingly, AQP5 differently impacted ROS levels in breast cancer cell lines. In MCF7 cells exposed to 40 µM H_2_O_2_, MCF7-AQP5 cells showed a significant increase in intracellular ROS compared to both control and pCMV6 cells. Conversely, SUM 159-AQP5 cells exhibited a decrease in ROS levels. As AQP5 is a peroxiporin, it is expected that higher expression of AQP5 would allow more H_2_O_2_ to enter the cell, increasing intracellular ROS in a milieu with elevated H_2_O_2_ concentrations, as observed in MCF7 cells. The inverse results in SUM 159-AQP5 cells suggest that H_2_O_2_ transport is tightly regulated. Molecular dynamic simulations revealed that AQP5’s gating mechanism involves spontaneous fluctuations that drive distinct open and closed conformations, with a tap-like mechanism at the cytoplasmic end regulated by His67, controlling water passage, while the selectivity filter at the extracellular end, modulated by His173, fine-tunes the flow rate [41]. Furthermore, AQP regulation of H_2_O_2_ gating involves selective permeability of barriers in the selectivity filter (SF) and NPA region of aquaporins, with H_2_O_2,_ encountering more restrictive barriers than water does [42]. The involvement of His173 in the selectivity filter and its interaction with Ser183 has been shown to play a key role in AQP5-facilitated H_2_O_2_ diffusion, contributing to cellular adaptation to oxidative stress [43]. While the regulation mechanism requires further exploration, our results suggest that SUM 159 cells demonstrate better regulation and adaptation to 40 µM H_2_O_2_.

Further, H_2_O_2_ treatment differentially regulated AQP5 and AQP3 expression in a cell-line-specific manner. Notably, *AQP5* mRNA levels decreased in SUM 159-AQP5 and SkBr-3-AQP5 cells on exposure to 10 µM H_2_O_2_ but returned to control levels at 40 µM H_2_O_2_, suggesting a transient downregulation in response to low oxidative stress, potentially as an adaptive mechanism. In contrast, *AQP3* expression in MCF7-AQP5 cells increased with rising H_2_O_2_ concentrations at the mRNA level, without a corresponding protein-level change. Such discrepancies between mRNA and protein levels are commonly observed [44] and are often attributed to stress responses and post-transcriptional regulatory mechanisms. Given that microRNAs (miRs) play a crucial role in post-transcriptional regulation, it is possible that certain miRs contribute to the observed discordance in AQP3 expression. While miRNA-mediated targeting of AQP3 has not been specifically reported in breast cancer, miR-mediated targeting of AQP3 by miR-185-5p, miR-874, and miR-124 has been shown to inhibit cell differentiation in various cancers [45].

The NRF2 signaling pathway is the main pathway activated as a response to oxidative stress and is often constitutively activated in various cancers, including breast cancer, contributing to therapy resistance [46]. Given its importance, we investigated whether overexpression of AQP5 influences the NRF2 signaling pathway in response to H_2_O_2_-induced oxidative stress. To assess this question, we analyzed the RNA expression of *NFE2L2* (*NRF2*) by qPCR and its protein levels by western blot; we performed corresponding testing for NRF2 repressor KEAP1 and the downstream targets AKR1B10 and HO-1. The findings indicate that AQP5 overexpression influences NRF2 signaling in a cell-line-specific manner in response to oxidative stress induced by H_2_O_2_. In MCF7 cells, AQP5 overexpression activated the NRF2/AKR1B10 axis following H_2_O_2_ treatment. This is particularly relevant given that AKR1B10 overexpression has been implicated in breast cancer cell adhesion, migration, and invasion via the integrin α5-mediated focal adhesion signaling pathway, a pathway that involves Rac1 [47], that was previously identified as a downstream signaling partner of AQP5, and that contributes to cell migration [48]. Thus, our findings suggest a potential link between AQP5, NRF2 activation, and pro-metastatic signaling in MCF7 cells. In SkBr-3 cells, AQP5 overexpression had a more modest effect, increasing NRF2 expression after treatment with 10 µM H_2_O_2_, but without significant changes in downstream targets or KEAP1 levels. In SUM 159 cells, transfection generally increased *NRF2* mRNA expression in both SUM 159-pCMV6 and SUM 159-AQP5 cells compared to control SUM 159 cells, regardless of H_2_O_2_ treatment. However, there was a notable increase in basal expression of the NRF2 downstream target HO-1 in SUM 159-AQP5 cells compared to its expression in control SUM 159, indicating that AQP5 may modulate HO-1 expression in SUM 159 cells, potentially enhancing cellular antioxidant capacity. Notably, we also observed increased *NRF2* expression, both under basal conditions and after exposure to 10 µM H_2_O_2_ in MCF7-pCMV6 cells, as well as an increase in NRF2 following treatment with 40 µM H_2_O_2_ in SkBr-3-pCMV6 cells along with an increase in HO-1 under basal and H_2_O_2_-stimulated conditions in MCF7-pCMV6 and SkBr-3-pCMV6 cells. These findings indicate that the empty plasmid influences the NRF2/HO-1 axis in MCF7 and SkBr-3 cells and affects NRF2 expression in SUM 159 cells, emphasizing the importance of appropriate controls in oxidative stress studies.

Finally, given their roles in cancer progression and the oxidative stress response, we investigated the impact of AQP5 overexpression and H_2_O_2_ treatment on PI3K/AKT signaling and FOXO transcription factors. The PI3K/AKT signaling pathway promotes tumor-cell proliferation, survival, cancer progression, and therapy resistance [11]. Moreover, PI3K/AKT signaling negatively regulates FOXO transcription factors [49], which, in turn, control antioxidant enzymes responsible for H_2_O_2_ detoxification, such as catalase, as well as cellular processes like the cell cycle, apoptosis, and metabolism, making them essential for the oxidative stress response [50].

AQP5 overexpression did not activate the PI3K/AKT signaling pathway in breast cancer cell lines, as evidenced by the lack of increase in levels of phosphorylated AKT and the pAKT/AKT ratio. Notably, AQP5 reduced total AKT expression in SkBr-3-AQP5 and MCF7-AQP5 cells, suggesting a possible inhibitory effect on AKT-driven signaling. In SUM 159-AQP5 cells, AQP5 increased levels of PTEN, a key negative regulator of PI3K/AKT signaling, following exposure to 40 µM H_2_O_2_, an effect also observed in control SUM 159 cells. Additionally, AQP5 decreased PI3K levels upon treatment with 40 µM H_2_O_2_ in SUM 159-AQP5 cells and restored PTEN expression in SkBr-3-AQP5 cells after treatment with 10 µM H_2_O_2_. However, these findings were influenced by fluctuations induced by the empty vector. Furthermore, transfection alone increased basal PI3K levels in MCF7 cells and decreased PTEN levels in SUM 159 cells, highlighting the importance of using proper experimental controls when investigating PI3K/AKT signaling modulation. Interestingly, our findings align with those of previous reports that PI3K signaling regulates PTEN expression through mammalian target of rapamycin (mTOR)-dependent translation, creating a feedback loop where PI3K activation induces PTEN to fine-tune pathway activation in response to stimuli [51]. These results suggest that in SUM 159 cells, AQP5 may regulate PI3K/AKT signaling by modulating PTEN, rather than by directly activating the pathway.

The involvement of AQP5 and FOXOs in the response to H_2_O_2_ showed cell-type-specific effects. In MCF7 cells, AQP5 had minimal impact on FOXO1 and FOXO3a expression, with only a slight, non-significant increase in FOXO1 levels upon H_2_O_2_ treatment, while H_2_O_2_ alone led to a reduction in *FOXO3a* levels in control cells. In SkBr-3 cells, AQP5 overexpression increased *FOXO3a* expression following H_2_O_2_ treatment, whereas transfection generally resulted in lower basal FOXO1 levels compared to those observed in control cells. The higher FOXO1 levels observed in control cells declined with increasing H_2_O_2_ concentrations. In SUM 159 cells, the modulation of FOXO1 and FOXO3a expression was only partially attributed to AQP5, with additional changes resulting from transfection. Specifically, AQP5 reduced FOXO1 levels at 10 µM H_2_O_2_, and the levels were restored on exposure to 40 µM H_2_O_2_. These findings suggest that AQP5 may have a dynamic role in regulating FOXO1 levels, with that role influenced by the severity of oxidative stress. Notably, the empty vector also influenced FOXO1 expression in SUM 159 cells, particularly under basal conditions, highlighting the importance of proper controls in experimental designs. Overall, AQP5 regulates FOXO expression in a cell-line-specific manner in response to oxidative stress, with the most notable effects observed in SkBr-3 and SUM 159 cells.

Our study demonstrates that AQP5 overexpression enhances sensitivity to H_2_O_2_-induced oxidative stress in breast cancer cell lines, resulting in significant reductions in cell viability at 40 µM H_2_O_2_ in SkBr-3 and MCF7 cells. In contrast, in SUM 159 cells, the reduced viability was attributed to the transfection process rather than to AQP5 itself. AQP5 modulated intracellular ROS levels differently across cell lines, increasing ROS levels in MCF7 cells and decreasing it in SUM 159 cells, although its direct activity was not assessed. Furthermore, AQP5 influenced various signaling pathways in a cell-type-specific manner, with effects including activation of the NRF2/AKR1B10 axis in MCF7 cells, increased basal HO-1 expression in SUM 159 cells, modulation of PTEN to regulate PI3K/AKT signaling in SUM 159 cells, and a reduction in AKT expression in SkBr-3 and MCF7 cells. A modest impact of AQP5 on FOXO1 and FOXO3a expression was also observed, with a decrease in FOXO1 levels in SUM 159 cells and an increase in FOXO3a mRNA levels in SkBr-3 cells. Notably, the empty plasmid influenced signaling pathways like NRF2/HO-1 in MCF7 and SkBr-3 cells, highlighting the importance of using proper controls when assessing AQP5’s role in oxidative stress. We also acknowledge that other downstream targets may be involved, an aspect which we plan to investigate further, and that testing at different time points could alter the outcomes. While our study focused on the acute response to H_2_O_2_, future research into prolonged exposure is necessary to explore how AQP5 may influence adaptation to H_2_O_2_ and contribute to resistance to H_2_O_2_-inducing therapies, as seen in MCF7 cells [52]. A limitation of this study is its in vitro setting, where exposure was restricted to H_2_O_2_ without cell−cell interactions. While H_2_O_2_ is the primary ROS involved in oxidative stress-related therapies, other oxidative stress byproducts may have a minor influence on treatment outcomes in vivo. Furthermore, extrapolating our findings and correlating H_2_O_2_ concentrations to in vivo systems remains challenging due to the difficulty of accurately measuring H_2_O_2_ in living organisms. Its short lifespan makes it undetectable by the time samples are analyzed, and freezing or homogenizing tissues further compromises measurement reliability. Consequently, oxidative stress is primarily assessed in vivo through biomarkers of oxidative damage [53].

In summary, our findings suggest that AQP5 overexpression in breast cancer cells under acute oxidative stress has cell-type-specific effects, with some modulation of key signaling pathways, including NRF2, PI3K/AKT, and FOXO, in response to H_2_O_2_ treatment. These results highlight the need for further research to better understand how AQP5 may influence the efficacy of and response to oxidative stress-inducing therapies, particularly in different breast cancer subtypes. Additionally, caution is needed when interpreting the data, as transfection itself can alter responses. Proper controls, including wild-type cells, are essential to account for these effects.

## 4. Materials and Methods

### 4.1. Cell Culture

For this study, we utilized three human breast cancer cell lines: MCF7 (hormone receptor (HR)-positive, including estrogen receptor (ER) and progesterone receptor (PR), and HER2-negative), SkBr-3 (HR-negative and HER2-positive), and SUM 159 (triple-negative, lacking ER, PR, and HER2). The cell lines were obtained from EACC (Porton Down, UK) or Elabscience (Vienna, Austria). They were cultured in Dulbecco′s Modified Eagle′s Medium (DMEM D6429, Sigma Aldrich, St. Louis, MO, USA) supplemented with 10% fetal calf serum (FCS F7524, Sigma Aldrich, St. Louis, MO, USA) and maintained in a humidified atmosphere with 5% CO_2_ at 37 °C. Once semiconfluent, the cells were trypsinized, counted, and seeded for subsequent treatments.

### 4.2. Establishing Stable AQP5-Overexpressing Cell Lines

**Figure 7:**
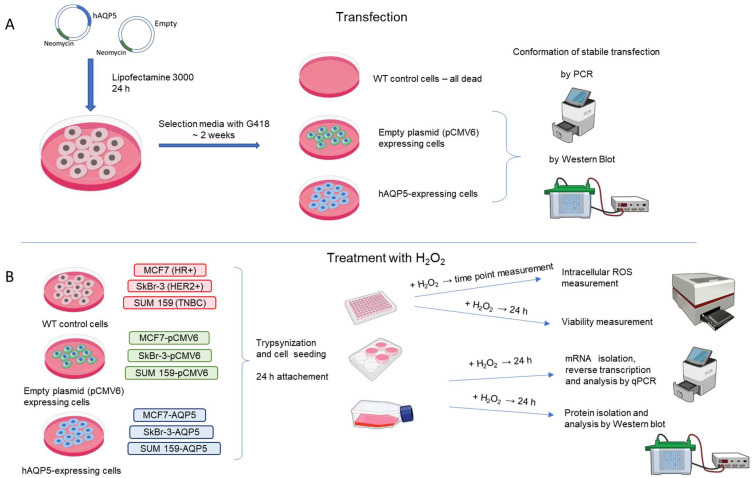
Experimental design. (**A**) Establishing cell lines stably expressing AQP5 or empty vector (pCMV6); (**B**) treatment with H_2_O_2_. Abbreviations: HR+—hormone receptor positive; HER2+ HER2-overexpressing cells; TNBC—triple-negative (hormone (estrogen and progesterone) receptor-negative and HER2-negative breast cancer.

Breast cancer cell lines (3 × 10^5^ cells per well) were seeded in six-well plates (TPP, Trasadingen, Switzerland), allowed to attach overnight, and transfected the following day with either the AQP5 (Myc-DDK-tagged)-Human-pCMV6 vector (RC206069) or the pCMV6-Entry vector (mock control, PS100001) (Origene, Rockville, MD, USA) using Lipofectamine 3000 (L3000001, Thermo Fisher Scientific, Waltham, MA, USA), following the manufacturer’s protocol. Transfected cells were cultured under G 418 selection (concentration selected from kill curve, Figure S1; G418-RO, Roche, Manheim, Germany) for at least two weeks before the experiments. Simultaneously, control cells without the plasmid were also exposed to G418 to confirm that all non-transfected cells were eliminated, ensuring that the remaining population consisted entirely of transfected cells. Transfection efficiency was verified via western blot and qPCR. A schematic representation of the experimental protocol is provided in Figure 7.

### 4.3. Cell Viability

For the cell-viability assay, 1 × 10^4^ cells per well were seeded into a 96-well plate (TPP, Trasadingen, Switzerland), allowed to attach for 24 h, and then exposed to various concentrations of H_2_O_2_ (0, 2.5, 5, 10, 20, 40, 70, and 100 µM). After an additional 24 h of incubation, cell viability was determined using the EZ4U MTT assay (BI-5000, Biomedica, Vienna, Austria) according to the manufacturer’s instructions. The resulting color change, indicating cell viability, was measured at 450 nm using a plate reader (EZ Read 2000, Biochrom, Cambridge, UK), with 620 nm as the reference wavelength.

### 4.4. ROS Measurement

Intracellular ROS levels were determined using 2′-7′-dichlorodihydrofluorescein diacetate (DCFH-DA D6883; Sigma-Aldrich, St. Louis, MO, USA). In living cells, DCFH-DA is deacetylated and subsequently oxidized by ROS, producing a fluorescent signal detectable at an excitation/emission wavelength of 500/529 nm. To investigate the effect of AQP5 on intracellular ROS production under H_2_O_2_ challenge, 1 × 10^4^ cells per well were seeded into a 96-well plate (TPP, Trasadingen, Switzerland), allowed to attach for 24 h, and incubated with DCFH-DA for one hour. After the dye was removed, the cells were treated with varying concentrations of H_2_O_2_. Fluorescence was measured at multiple time points using an Infinite 200 PRO microplate reader (Tecan, Männedorf, Switzerland), and the values were normalized to the cell-viability readings from the same plate.

### 4.5. mRNA Extraction and qPCR Measurement

After 24 h of H_2_O_2_ exposure, total RNA was extracted using TRIzol (15596026, Thermo Fisher Scientific, Waltham, MA, USA) according to the manufacturer’s instructions. RNA purity and concentration were assessed spectrophotometrically using the NanoPhotometer^®^ N60 (Implen GmbH, München, Germany). Subsequently, 1 µg of RNA was reverse-transcribed into cDNA using the High-Capacity cDNA Reverse Transcription Kit (4368814, Thermo Fisher Scientific, Waltham, MA, USA) following the manufacturer’s protocol on an Eppendorf 5331 MasterCycler Gradient Thermal Cycler (Eppendorf, Hamburg, Germany).

Quantitative analysis of transcripts of AQP3, AQP5, NFE2L2, FOXO1, FOXO3a, and the housekeeping gene B2M was performed using SYBR Green chemistry. Primers were designed as detailed in Table 1. Each reaction mixture contained 10 µL of SsoAdvanced Universal SYBR Green Supermix (1725274, Bio-Rad Laboratories, Hercules, CA, USA), 8 µL of deionized water, 0.5 µL of a forward and reverse primer mix (5 µM each), and 1.5 µL of cDNA template. The amplification protocol included an initial incubation at 95 °C for 2 min, followed by 40 cycles of 95 °C for 15 s and 62 °C for 30 s. Relative gene-expression levels were calculated using the 2^−ΔΔC(T)^ method [54].

### 4.6. Protein Extraction and WB Analyses

After 24 h of H_2_O_2_ exposure, total proteins were extracted using RIPA buffer supplemented with protease and phosphatase inhibitors (Halt™ Protease and Phosphatase Inhibitor Cocktail 78440; Thermo Fisher Scientific, Waltham, MA, USA). Protein concentrations were determined using the Bradford method [55]. Aliquots containing 10–15 µg of protein were separated by SDS-PAGE on a 9% resolving gel. Proteins were then transferred to a nitrocellulose membrane (Roti-NC 0.2 µm; Carl Roth, Karlsruhe, Deutschland), stained with Ponceau S, and scanned. The membrane was blocked with 5% nonfat dry milk, washed, and incubated overnight with primary antibodies (listed in Table 2). After washing, the membrane was incubated with HRP-conjugated secondary antibodies, either anti-rabbit IgG (1:2000, CST-7074, Cell Signaling Technology (CST), Danvers, MA, USA) or anti-mouse IgG (1:4000, CST-96714S, CST). Signals were visualized using the SuperSignal™ West Pico PLUS Chemiluminescent Substrate (34580, Thermo Fisher Scientific, Rockford, IL, USA), and chemiluminescence was detected with the Alliance 4.7 Digital Imaging System (Uvitec, Cambridge, UK). Signal quantification was performed using Nine Alliance software (Uvitec), with protein expression normalized to total protein levels based on Ponceau S staining (P7170, Sigma Aldrich, St. Louis, MO, USA).

### 4.7. Statistical Analysis

All experiments were repeated at least three times, with technical triplicates included where relevant. Results are expressed as the mean ± standard deviation (SD). Statistical analysis was performed using Prism GraphPad 8.0 (GraphPad Software, San Diego, CA, USA) with two-way ANOVA, followed by Tukey or Bonferroni post hoc tests. *p*-values < 0.05 were considered statistically significant.

## Figures and Tables

**Table 1:** Primer sequences used for qPCR analysis.

Gene of Interest	Forward	Reverse
NRF2	GCTATGGAGACACACTACTTGG	CCAGGACTTACAGGCAATTCT
AQP3	GGGCTGTATTATGATGCAATCTGG	GTCCAGAGGGGTAGGTAGCA
AQP5	CCCGCTCACTGGGTTTTCT	GTCCTCGTCAGGCTCATACG
FOXO1	GTCCTACGCCGACCTCAT	ACTTGCTGTGTAGGGACAGAT
FOXO3a	GGGGAACTTCACTGGTGCTA	GTTTGAGGGTCTGCTTTGCC
B2M	TGTCTTTCAGCAAGGACTGGT	ACATGTCTCGATCCCACTTAAC

Abbreviations: NRF2, nuclear factor erythroid 2–related factor 2; AQP3, aquaporin 3; AQP5, aquaporin 5; FOXO1, forkhead box class O 1; FOXO3a, forkhead box class O 3a; B2M β2 microglobulin.

**Table 2:** Primary antibodies used for western blot.

Antibody	Catalog Number	Supplier	Dilution
Anti-NRF2 (Rabbit)	CST-12721	Cell Signaling Technology (CST)	1:1000
Anti-PI3K (Rabbit)	CST-4249	Cell Signaling Technology (CST)	1:1000
Anti-p-AKT (Rabbit)	CST-4060	Cell Signaling Technology (CST)	1:1000
Anti-AKT (Rabbit)	CST-4691	Cell Signaling Technology (CST)	1:1000
Anti-KEAP1 (Rabbit)	CST-8047	Cell Signaling Technology (CST)	1:1000
Anti-HO-1 (Rabbit)	CST-26416	Cell Signaling Technology (CST)	1:1000
Anti-PTEN (Rabbit)	CST-9188	Cell Signaling Technology (CST)	1:1000
Anti-FOXO3a (Rabbit)	CST-2497	Cell Signaling Technology (CST)	1:1000
Anti-FOXO1 (Mouse)	CST-2880	Cell Signaling Technology (CST)	1:1000
Anti-AQP3 (Mouse)	sc-518001	Santa Cruz Biotechnology	1:200
Anti-AQP5 (Mouse)	sc-514022	Santa Cruz Biotechnology	1:200
Anti-AKR1B10 (Rabbit)	ab96417	Abcam	1:10,000

Abbreviations: PI3K, phosphoinositide 3-kinases; pAKT, phosphorylated protein kinase B; AKT, protein kinase B; KEAP1, Kelch-like ECH-associated protein 1; HO-1, heme oxygenase 1; PTEN, phosphatase and tensin homolog; AKR1B10, aldo-keto reductase family 1 member B10.

## Data Availability

The data are contained within this article.

## Supplementary Materials

The following supporting information can be downloaded at: https://www.mdpi.com/article/10.3390/ijms26073243/s1.
