# Supplementary material for: The Influence of AQP5 on the Response to Hydrogen Peroxide in Breast Cancer Cell Lines
Source: Int J Mol Sci. 2025 Mar 31;26(7):3243. doi: 10.3390/ijms26073243 (PMC11989815; doi:10.3390/ijms26073243)
Supplement: Supplementary file 1 [file ijms-26-03243-s001.zip › ijms-3482769-supplementary.pdf]

Supplementary

The influence of AQP5 in response of breast cancer cell lines to hydrogen-peroxide

Ivan Lučić, Monika Mlinarić, Ana Čipak Gašparović* and Lidija Milković*

1. **G418 kill curve**

We performed a G418 kill curve to determine the optimal G418 concentration for selecting breast cancer cell lines with stable overexpression of AQP5 or pCMV6 (mock control). After exposing the cell lines to increasing concentrations of G418 for 10 days, cell viability was assessed using the MTT-based EZ4U test, and 500 μg/mL G418 was chosen as the optimal concentration.

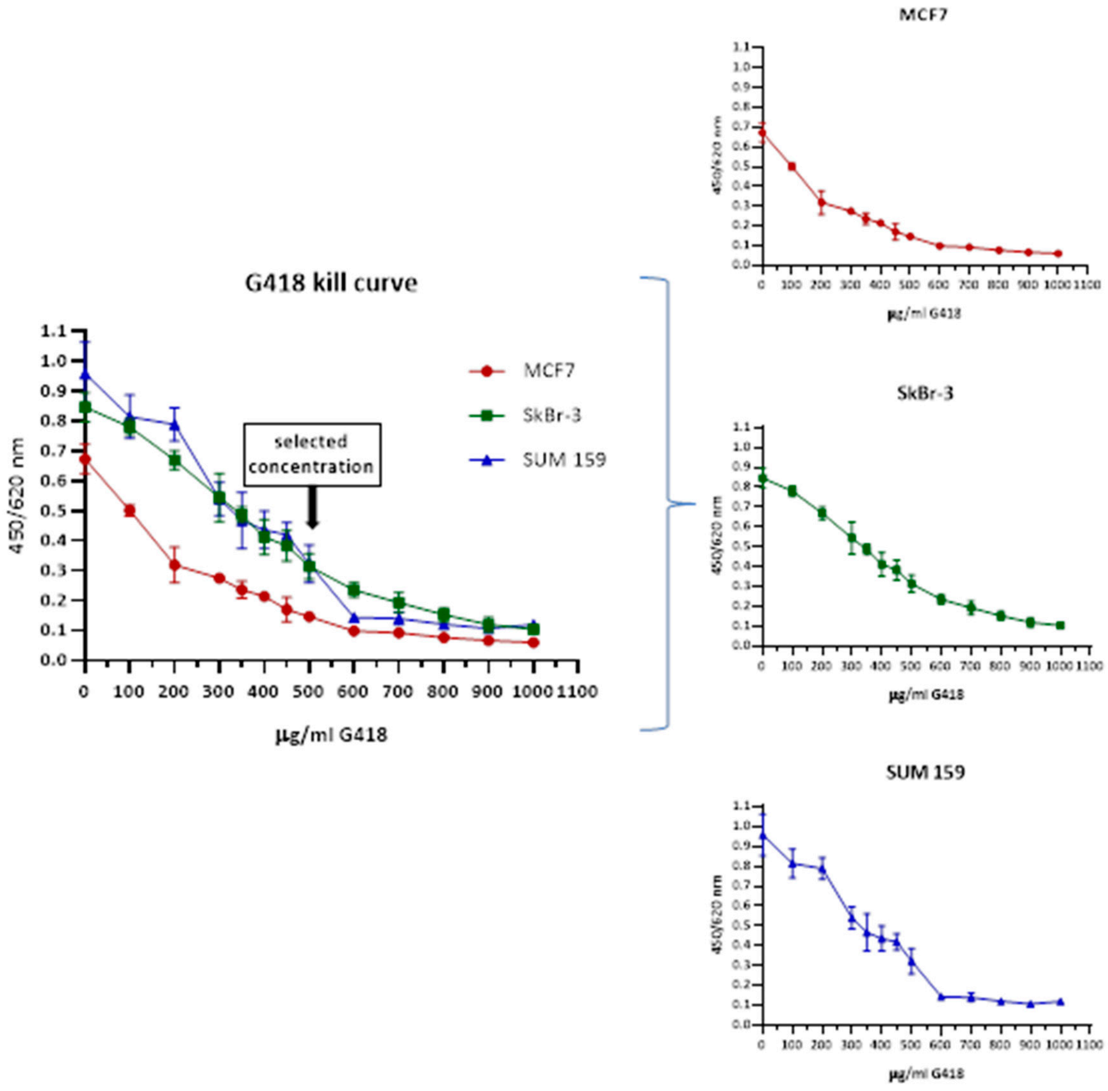

Figure S1. Selection of the G418 concentration for stable transfection

2. **Intracellular ROS measurement upon addition of 10 and 40 µM H_2O_2**

We evaluated intracellular ROS levels under basal conditions and following exposure to 10 µM and 40 µM H_2O_2 over time. Within 15 minutes, 40 µM H_2O_2 significantly increased ROS levels in all cells compared to their basal levels, with levels continuing to rise over time. Additionally, 10 µM H_2O_2 induced a ROS increase after 1 hour in AQP5-overexpressing and pCMV6 MCF7 and SkBr-3 cells ($p < 0.01$) and after 2 hours in all MCF7 and SkBr-3 cells ($p < 0.05$). In SUM 159 cells, ROS levels increased only in the control group after 1 and 2 hours ($p < 0.01$) and not in SUM 159-AQP5 or SUM 159-pCMV6 cells.

Interestingly, AQP5 had differing effects on ROS dynamics in MCF7 and SUM 159 cells. In MCF7-AQP5 cells, ROS levels increased significantly ($p < 0.0001$) 2 hours after the addition of 40 µM H_2O_2. Conversely, in SUM 159-AQP5 cells, ROS levels decreased compared to control and pCMV6 cells, starting at 30 minutes ($p < 0.01$).

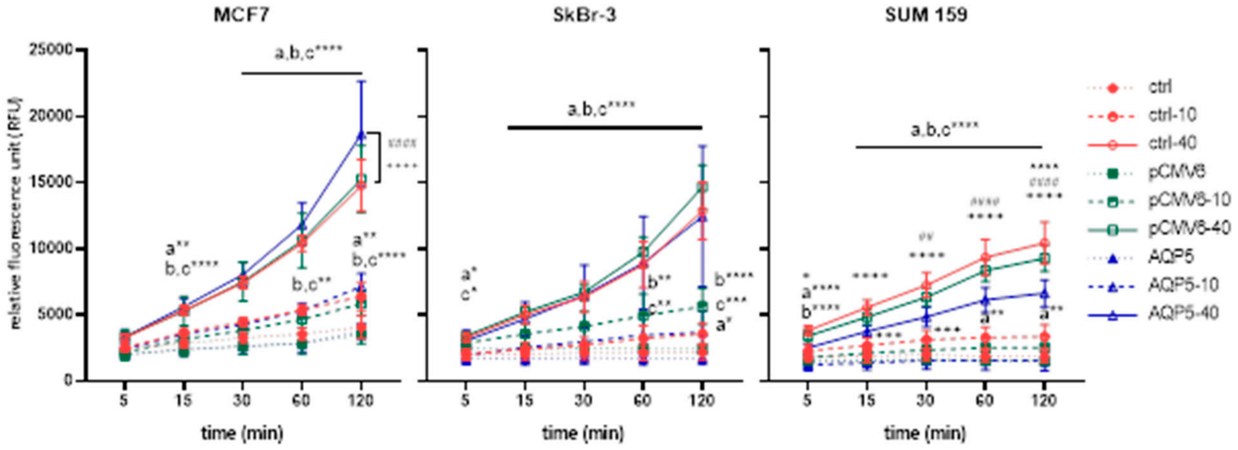

Figure S2. Levels of intracellular ROS (basal, upon 10 and 40 µM H_2O_2) during exposure time

The results are expressed as means ± SDs, n = 3. (a,b,c*) represents the difference between treatments and their control for: a - control cells; b – pCMV6 cells; c – AQP5 overexpressing cells. (*) represents the difference between control vs pCMV6 cells for the same treatment; (#) represents the difference between pCMV6 vs AQP5 cells for the same treatment; (+) represents the difference between control vs AQP5 cells for the same treatment. a*, b*, c* or *or+ or# $p < 0.05$; a**, b**, c** or **or++ or## $p < 0.01$; a***, b***, c*** or ***or+++ or### $p < 0.001$; a****, b****, c**** or ****or++++ or#### $p < 0.0001$.

3. **Comparison of protein expression of NRF2, KEAP1, and AQP5 in breast cancer cell lines by Western blot**

Western blot analysis of AQP5, NRF2, and KEAP1 revealed notable differences among the cell lines. The highest levels of AQP5 were observed in SkBr-3 cells, while MCF7 and SUM 159 cells exhibited similar levels of expression. This finding aligns with the qPCR data (Fig. 3), which showed nearly 10-fold higher AQP5 expression in SkBr-3 cells compared to SUM 159 and MCF7 cells.

Additionally, NRF2 and KEAP1 expression levels were lower in SkBr-3 cells compared to MCF7 and SUM 159 cells.

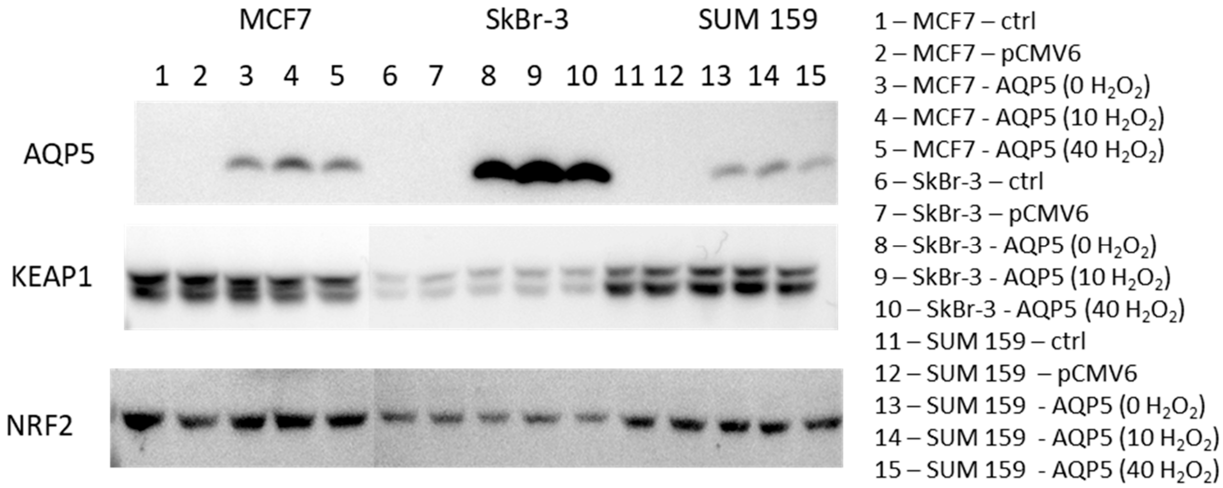

Figure S3. Comparison of protein expression of AQP5, NRF2, and KEAP1 in breast cancer cell lines.
